# Corneal Treatment, Repair, and Regeneration: Exosomes at Rescue

**DOI:** 10.3390/pharmaceutics16111424

**Published:** 2024-11-07

**Authors:** Brooke T. Robbins, Kate A. Montreuil, Neloy Kundu, Prashant Kumar, Vibhuti Agrahari

**Affiliations:** 1 Department of Pharmaceutical Sciences, College of Pharmacy, University of Oklahoma Health Sciences Center, Oklahoma City, OK 73117, USA; 2 Graduate College, University of Oklahoma Health Sciences Center, Oklahoma City, OK 73117, USA; 3 Vaccine Analytics and Formulation Center, Department of Pharmaceutical Chemistry, University of Kansas, Lawrence, KS 66047, USA; prashant.kumar@ku.edu

**Keywords:** corneal healing cascade, hydrogels, immunogenicity, regeneration, restoration, stem cells

## Abstract

Exosomes are extracellular vesicles within the nanosized range that play roles in intercellular communication and thus have certain biological activities. The secretory signaling communication mechanism is an efficient way of exchanging information between cells and has been investigated as nature’s therapeutic drug carriers. This review will summarize the potential of exosomes as therapeutic tools and drug delivery vehicles for corneal pathologies. The cornea is an avascular ocular tissue, and its healing is a complex process including cell death and migration, cell proliferation and differentiation, and extracellular matrix remodeling. Here, we discussed the structure, barrier, phases, and healing cascade of cornea. We briefly reviewed the immunogenicity and toxicity of exosomes and role of exosomes in preserving cornea. Additionally, we provided combining exosome strategies with hydrogels, gene and stem cells therapy focused on corneal treatment, repair, and regeneration.

## 1. Introduction

Vision impairment occurs when an eye condition affects the visual systems and functions. According to the WHO 2019 global report, 2.2 billion people are experiencing some ocular issues. Among ocular diseases, corneal defects and illnesses are the third leading cause of vision impairment worldwide. Disorders like corneal scarring, haze, dry eye, keratitis, keratoconus, corneal dystrophies, herpes infection, cicatrizing conjunctivitis, Surfer’s eye, fibrosis, Stevens–Johnson syndrome, and iridocorneal endothelial syndrome are common corneal conditions that affect normal vision [1]. Corneal opacities, which can be caused by a range of factors like trauma, infections, hereditary conditions, and previous surgeries, contribute significantly to visual impairment. The global burden of corneal opacities was responsible for 1.9 million cases of moderate to severe visual impairment in 2019, and they were reported as the primary cause of blindness [2]. It has been established physiologically that corneal healing involves complex pathways. Thus, varying mechanisms of actions are often involved to support corneal recovery, repair, and regeneration. Particularly, for the corneal wound-healing mechanism, several approaches have been identified, including gene therapy and biologic therapeutics, and there is a plethora of drug classes employed by ophthalmologists to control eye pain, combat inflammation, and treat ocular surface disease. However, the complexity of five-layered corneal tissue still needs patient-friendly and effective treatments. Thus, we explored the potential of exosomes as therapeutics for repair, regeneration, and treatment of cornea.

Exosomes, a class of tiny extracellular vesicles (EVs), have been used as a suitable delivery vehicle owing to their inherent function in intracellular communication and biocompatibility [3]. EVs are lipid-bound, small cellular containers secreted by nearly all cell types. Subcategories of EVs include apoptotic bodies, microvesicles, and exosomes, which are differentiated based on size and pathway of origin [4,5]. Exosomes, being the smallest subtype of EVs, range in diameter from only 30 to 150 nanometers (nm) [6]. Despite their small size, the nanovesicles contain fundamental building blocks of cells, including nucleic acids, proteins, and lipids from their derived parent cell. Their main feature and most understood function to date is their role as signaling molecules through the transfer of stored genetic information, serving as a biomarker for disease, as well as being proposed therapeutics by themselves (non-drug- or gene-loaded) [7]. Due to those described roles of exosomes, they have shown promise as a non-invasive treatment for corneal disease. In this article, we will review the use of exosomes to rescue cornea, which includes the source of exosomes, therapeutic effects, and limitations to clinical use. Overall, this succinct review will provide concise summaries of cornea anatomy, physiology, wound-healing mechanism, biogenesis, and immunogenicity of exosomes and exosome-based approaches for corneal repair, regeneration, and treatment.

## 2. Cornea Structure and Barrier

**Figure 1:**
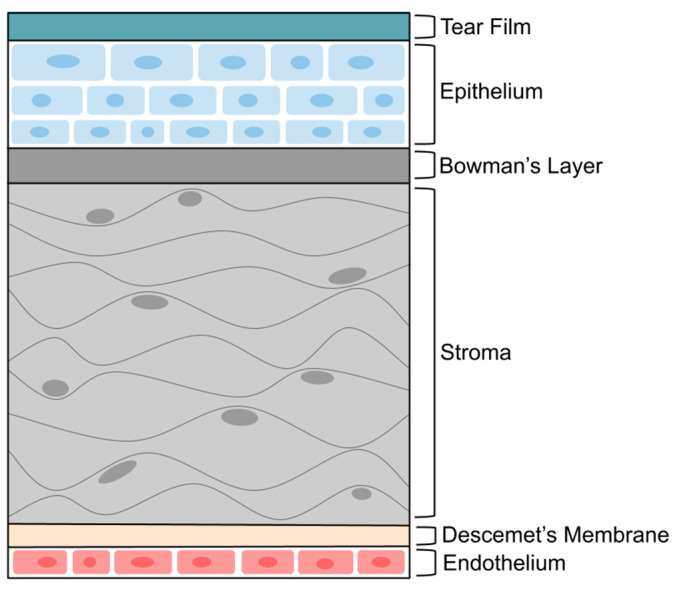
Layers of cornea (prepared using Google drawings).

In recent years, ophthalmic drug delivery research has focused on improving the ability of drugs to penetrate the many biological barriers present in the eye [8]. These barriers, the most notable of which are the tear film and corneal barrier, represent a special challenge for topically administered ophthalmic drugs [9]. The tear film is the first barrier encountered by any drug applied topically to the eye; blinking and tear dilution cause rapid clearance and reduced bioavailability of drugs administered via this route. The tear film is composed of three layers: lipid, aqueous, and mucin; the mucin layer is the deepest and is most closely located to both the cornea and the conjunctiva [4]. As illustrated in Figure 1, the cornea itself is composed of an epithelial layer, the Bowman membrane, the corneal stroma, the Descemet membrane, and the corneal endothelium [10]. Each layer provides some type of anatomical or physiological support that is necessary to the function of the cornea; together, all these layers form a mechanical barrier that hinders microbial and foreign body penetration into the eye. Cornea protects the internal eye structures and is responsible for two-thirds of the refractive power of the eye by focusing light correctly on the retina. The corneal composition can be broken down into the cellular components: epithelial cells, keratocytes, and endothelial cells and the acellular components: collagen and proteoglycans. The permeability barriers of the cornea are the epithelium and stroma. The corneal structure is critically dependent on the dynamic maintenance of corneal hydrostasis to ensure proper hydration.

Starting at the outermost layer of the cornea, the corneal epithelium is transparent; non-keratinized; stratified; and is comprised of layers of squamous, basal, and wing cells [11]. These epithelial cells are connected to one another via tight junctions that prevent the entry of foreign materials or organisms into the cornea [10]. The epithelial layer is also the most anteriorly located layer of the cornea and is similar to the Descemet membrane in its ability to regenerate after disease, trauma, or desquamation. Structurally, the epithelial layer forms the outermost surface of the eye but is limited in its ability to grow or expand into deeper parts due to the basement membrane [11]. The main functions of the corneal epithelium include physical protection, refraction, radiation protection, tear stabilization, barrier protection, and mucous production [12]. Bowman’s layer is located on the posterior side of the basement membrane; the posterior side of the basement membrane projects collagen fibrils that interact with the surface of the stroma [13]. This layer does not act as a barrier to foreign objects, microbes, or drugs and does not regenerate post-injury. The function of Bowman’s membrane has often been a topic of debate, with many suggesting that it possesses some sort of barrier function; however, it is not clear what its main function is based upon the available literature. It is likely that Bowman’s layer is involved in or maintained by epithelial–stromal interactions [13]. The stroma is the largest layer of the cornea and is characterized by its transparency, avascularity, and other physical properties; it is responsible for providing the cornea with structural support. Keratocytes, which are most localized to the stroma, are responsible for the secretion of the extracellular matrix (ECM) [14]. The ECM is used to maintain hydration in the eye, modulate angiogenesis, and regulate intraocular pressure [15]. Keratocytes also cause a backscatter reduction of light, reducing the transparency of the cornea and producing soluble protein “crystallins”, which reduce the backscatter of light and maintain corneal transparency [16]. The Descemet membrane is the basement membrane of the corneal endothelium and anchors the endothelium to connective tissues. It is comprised of collagen and glycoproteins and lacks elasticity despite being mostly resistant to trauma, damage, or disease [4]. The Descemet membrane is involved in physiologic activities that maintain the corneal structure and homeostasis; it allows for molecular and nutrient transport, regulates corneal hydration, and maintains transparency [17]. The endothelium, which secretes the ECM forming the Descemet membrane, is comprised of a single cell layer, all of which are hexagonal in shape. The endothelium is responsible for maintaining the dehydrated state of the stroma that is necessary for transparency and unobstructed vision; this dehydrated state is maintained through ionic pumps on the endothelium’s basolateral plasma membranes [12].

## 3. Corneal Wound-Healing Processes

**Figure 2:**
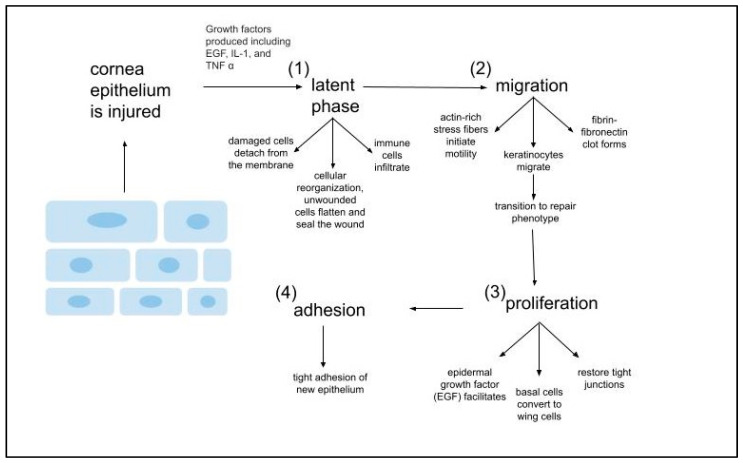
Four phases of epithelial wound-healing (prepared using Google drawings).

**Figure 3:**
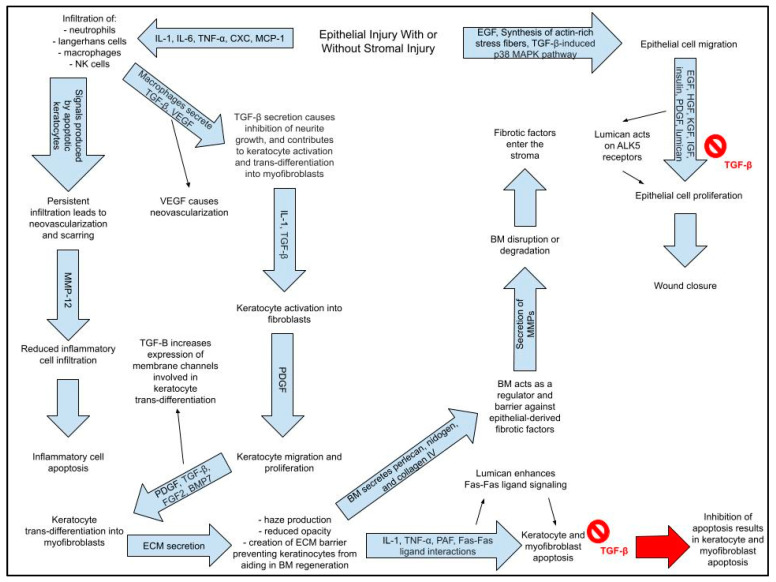
Cascade during corneal injury and inflammatory responses (prepared using Google drawings).

Corneal wound-healing is a critical process for restoring corneal integrity and maintaining vision after corneal injury/trauma. The aggressive inflammatory responses following ocular injuries and bacterial invasion often tend to impair corneal re-epithelization, which results in the loss of corneal transparency and impairment of vision [18]. When the cornea sustains an injury due to trauma, chemical attack, environmental damage, or infection to the eye, a wound-healing cascade is initiated to rapidly address the damage [1]. Most corneal injuries are superficial and occur at the most anteriorly located layers of the cornea: the epithelium and its basement membrane. The epithelium is the only layer of the cornea that possesses regenerative ability for both normal maintenance and injury. Corneal epithelium wound-healing is a multi-stage process described in Figure 2, which is separated into four phases: the lag or latent phase, the migratory, proliferation, and assembly of adhesion structures [19]. The phases occur chronologically but may overlap in time and cascade of inflammatory responses after cornel integrity is broken, described in Figure 3 and discussed in this section.

The latent phase of corneal epithelial wound-healing is responsible for conducting cellular reorganization. The cellular reorganization increases cellular motility and initiates the migratory phase so that re-epithelialization may take place [19]. During the migration phase, cell layers extend over the wound to maintain the cornea’s integrity; the migration of these corneal epithelial cells to the wound site is dependent on the synthesis of actin-stress fibers [20]. The purpose of the proliferative phase is to repopulate the wound site with corneal epithelial cells; both limbal and peripheral epithelial cells exhibit enhanced proliferation rates during this period [21]. The last phase of corneal epithelial wound-healing involves the formation of adhesion structures; cells on the epithelium’s basement membrane form hemidesmosomal attachments that secure epithelial structures to underlying connective tissues [20]. The formation of these adhesion structures results in wound closure. During the early phases of corneal epithelial wound-healing, many types of growth factors and/or cytokines are produced, such as epidermal growth factor (EGF), keratinocyte growth factor 1 (KGF-1), and hepatocyte growth factor (HGF) [19,22]. Stromal injury almost always coincides with injuries to the epithelium and basement membrane because of the stroma’s more posterior location. Within a few minutes of stromal injury taking place, keratocytes undergo apoptosis and/or necrosis that is triggered by an influx of interleukin-1 (IL-1) and tumor necrosis factor-alpha (TNF-α); the keratocytes adjacent to the injury proliferate and migrate [18,23]. These keratocytes will later be activated to become fibroblasts and will eventually transform into myofibroblasts [18,24]. Transforming growth factor (TGF)-β1, TGF-β2, and platelet-derived growth factor (PDGF) are responsible for the development of mature, alpha-smooth muscle actin (SMA)+ myofibroblasts; this myofibroblast phenotype secretes disordered ECM that contributes to corneal scarring, corneal opacity, stromal fibrosis, visual field defects and/or loss of visual acuity [24]. The ECM also acts as a physical barrier to keratocytes, preventing them from assisting in regeneration of the epithelial basement membrane. Myofibroblasts may also be responsible for the inhibition of neurite outgrowth; TGF-β contributes to sensory dysfunction via involvement in a pathway that inhibits nerve regeneration [18,25].

The Descemet membrane is similar to the stroma in that it lacks the ability to regenerate; because of this, keratoplasty is the only available method for healing wounds to the Descemet membrane and the corneal endothelium. It is thought that the endothelium’s reduced ability to self-heal can be contributed to lower rates of cell proliferation; thus, the endothelium relies on cellular migration and cell spreading to address any injuries [21]. Cell migration is stimulated by several ECM proteins, growth factors, and cytokines. As endothelial cells migrate, they begin to exhibit a fibroblastic phenotype through the expression of alpha-smooth muscle actin and the loss of tight junction proteins; the fibrotic changes produced by this transition can contribute to the formation of a retrocorneal fibrous membrane [21].

Why stromal regeneration and EBM restoration are important: ultrastructural studies have demonstrated that defective epithelial basement membrane (EBM) regeneration after injury to the cornea underlies the development of myofibroblasts that become established in the anterior stroma. These myofibroblasts often persist for months or years after the injury and are responsible for opacity. Therefore, full regeneration of the stroma is essential to maintain the transparency and to restore the EBM [18]. Since the corneal stroma is avascular and has a low keratocyte density, it could be healed by the exosomes, while subjected to in-depth investigations. The steps of a stromal wound-healing cascade are explained separately in Box 1 and explained with help of nine steps.

Box 1 **Steps of a stromal wound healing cascade.**

(1) Stromal injury
  (a) Stromal injury involves epithelial injury.
  (b) Epithelial injury + damage to basement membrane → stromal haze
    (i) Stromal haze → scarring, opacity → reduced transparency
  (c) Epithelial Healing Cascade
    (i) Upon injury, epithelial cells proliferate.
    (ii) Actin-rich stress fibers initiate migration to the wound
    (iii) Generation of adhesion structures that anchor the regenerated epithelium to the underlying connective tissue.
  (d) Epithelial healing cascade is regulated by GFs and cytokines, which primary function is to mediate interactions corneal epithelium and stroma via the basement membrane. These interactions lead to keratocyte apoptosis, keratocyte activation, and keratocyte trans-differentiation into myofibroblasts.
(2) IL-1, TNF-α, PAF, and Fas–Fas ligand interactions cause immediate apoptosis of keratocytes beneath corneal epithelium within the zone of injury.
  (a) Apoptosis causes infiltration of inflammatory cells via release of IL-1, IL-6, TNF-α, CXC chemokines, and MCP-1 (macrophage chemotactic protein-1) released from epithelial and stromal cells
(3) Some keratocytes undergo necrosis.
(4) Other keratocytes near the area of necrosis/apoptosis proliferate, migrate, and undergo activation into fibroblasts.
(5) Fibroblasts have the potential to transform into myofibroblasts.
(6) Fibroblasts and myofibroblasts secrete a provisional matrix scaffold.
(7) Epithelial and stromal cells secrete chemokines, which triggers an influx of inflammatory cells;
  (a) Inflammatory cells clear apoptotic and necrotic (#2 and #3) debris.
(8) Epithelial cells, inflammatory cells, fibroblasts, and myofibroblasts secrete collagenolytic metalloproteinases that cause stromal repair and remodeling
(9) Resorption of abnormal ECM and apoptosis or reversal of myofibroblast phenotype restores normal form and function of the stroma.

## 4. Exosomes for Preserving Cornea

**Figure 4:**
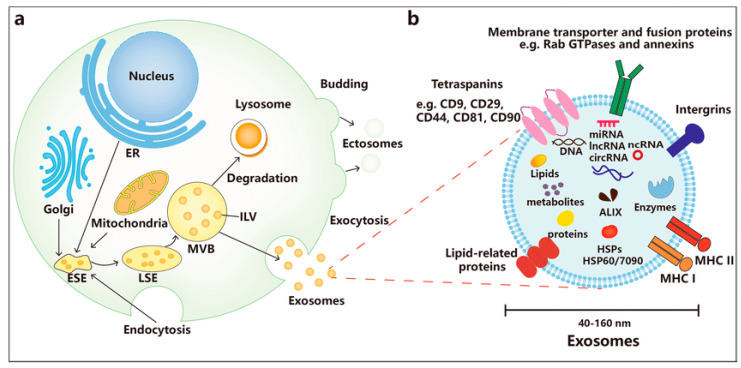
Biogenesis of MSC-derived exosomes. (**a**) comprises of endocytosis, MVB formation, and exosomes secretion into the extracellular environment through merging with the plasma membrane. (**b**) represents contents and characteristics of exosomes carrying a variety of substances such as proteins, lipids, nucleic acids, metabolites [26].

The vast majority of cells constantly discharge EVs to the extracellular environment to engage in different biological functions. There are two distinct biogenesis processes for EVs: (1) created naturally by breaking from the plasma membrane; (2) generated when the multivesicular body (MVB) fuses with the plasma membrane. Exosome biosynthesis is a continuous, endosomal-dependent cytological process [26], illustrated in Figure 4. While the initial endosomes created by cellular membrane invagination develop into late endosomes, intraluminal vesicles (ILVs) gather in the lumen. Specific-sorted ILVs, including lipids, proteins, and cytosol, are produced by internal splitting of the initial endosomal membrane. Multivesicular endosomes (MVEs), often called MVBs, are the terms used to describe late endosomes that include ILVs. The MVB in cells has two possible outcomes. These include MVB fusions with lysosomes, which destroy what they carry, and MVB fusions with the membrane of the plasma cell, which discharge ILVs into the external milieu as exosomes [7,26]. Chiefly, they are mediators of cell-to-cell communication through the transport of signaling molecules. Pertaining to the cornea, exosomes are being studied as non-invasive, natural drug delivery systems for cornea wound-healing, repair, and stromal regeneration. Currently, exosome drug delivery systems have been investigated as alternatives to surgical corneal transplants. In cornea, exosomes mediate communication between the epithelium, stroma, and endothelium. A study analyzed and characterized the production of exosomes by different cell types in the human cornea using exosome biomarkers: CD9, CD63, and CD81, where CD63 is the most used biomarker. The uptake of exosomes enriched with Human corneal epithelial cells (hCECs), corneal fibroblasts (hCFs), corneal endothelial cells (hCEnCs) was monitored [27].

## 5. Immunogenicity and Toxicity of Exosomes

The eye, an immune-privileged organ, can typically withstand the introduction of antigens or other foreign materials without incurring a compensatory immune response. This evolutionary adaptation protects the eye from damage caused by inflammation, scarring, etc. The role exosomes play in immunologic responses has been studied extensively; although, much of this research has focused on the immunomodulatory effects they may exert in cancer. Little research has been conducted regarding the potential toxic effects of exosomes on ocular tissues [28,29]. Exosomes derived from human sources are natural, nanosized, intercellular communication systems and are normally non-immunogenic and do not typically illicit a direct immune response. Most studies examining the effects of exosomes as a potential treatment for corneal disease and injury have found that they tend to inhibit undesirable immune responses, such as inflammation, scarring, fibrosis, and so on. However, this does not mean that exosomes have no immunogenic potential. Research has shown that exosomes sourced from human sources can elicit inflammatory responses in animal models [30]. This finding suggests a need for further research on the immunogenicity of exosomes when used in cross-species models. However, it has long been assumed that, due to exosomes’ inability to accumulate for long-term periods in tissues and organs, they exhibit minimal toxic effects [31]. This characteristic can be attributed to exosomes’ lack of systemic toxicity, but that does not mean that they are not able to exert local toxic effects. The local toxic effects, however, seem to only be exerted by exosomes that have been modified to encapsulate a potentially toxic substance or biologic molecule, by exosomes with modified protein content or cellular surface proteins, or by exosomes that are secreted from damaged or diseased cells [32]. The encouraging results from kidney allograft survival [33] and improved islet transplantation [34] suggest that exosomes from specific immunosuppressive cell populations serve as a potentially effective tool to promote immune tolerance in corneal graft survivals.

## 6. Exosomes Combined with Other Therapeutic Approaches

Exosomes are being recognized for their potential as delivery vehicles for a wide array of therapeutics, including hydrogels, gene delivery, biologic drugs, secretome from stem cells, etc. Table 1 summarizes several investigations on exosomes-based cornea treatment.

### 6.1. Exosomes with Hydrogels

Hydrogels are crosslinked polymeric networks exhibiting hydrophilic interactions with water molecules. Their hydrophilic and flexible properties make them an ideal drug delivery system and have been used to administer exosomes both in vitro and in vivo to ocular tissues [35]. Hydrogels offer several advantages, including adjustable mechanical behavior, cytocompatibility, and optical characteristics that make them suited for corneal regeneration and restoration [36]. Various kinds of biological and synthetic hydrogels, such as collagen/gelatin/alginate [37], collagen/hyaluronic acid [38] chitosan [39], and collagen/polyethylene glycol [40,41], have been used for corneal wound-healing [42]. There are three main strategies to incorporate exosomes into hydrogels [43]: (a) incorporate exosomes into the polymer solution before adding crosslinkers to the gel composite; (b) using a swelling/“breathing” approach, physically insert exosomes into pre-formed hydrogels; (c) combine exosomes and polymers, along with crosslinkers in real time for in situ gelation. Hydrogels are designed to dissolve when epithelial cells glue over the exposed stroma, preventing vision from being obstructed for an extended length of time. If the undamaged corneal epithelium needs to be protected from the start of medication, the exosome-loaded film/hydrogel shall be inserted intrastromally [44]. Tang et al. [45] developed an exosome-loaded thermosensitive hydrogel to regenerate the corneal epithelium and stroma. The exosomes used were derived from induced pluripotent stem cell-derived mesenchymal stem cells (iPSC-MSCs) and were combined with a chitosan-based, thermosensitive hydrogel that exhibited matrix-type release kinetics. The group’s in vivo study determined that the exosome-loaded thermosensitive hydrogel downregulates expression of the mRNA encoding collagens produced in the stroma, thus preventing ECM deposition during or after corneal injury. Through this mechanism, the hydrogel effectively reduces scarring, leading to corneal opacity, and accelerates the overall healing process. Additionally, the exosomes derived from iPSC-MSCs contained miR-432-5p, which is involved in the suppression of a modulator involved in the biosynthesis of collagen, further reducing ECM deposition and the risk for corneal opacity in their in vivo rat model [45]. Sun et al. [46] recently demonstrated that a miRNA-rich exosome-loaded thermosensitive hyaluronic acid-based hydrogel can promote corneal epithelial cell (CEC) migration and repair both in vitro and in vivo. Exosomes from adipose-derived mesenchymal stem cells were loaded into a functionalized, DEGMA [di(ethylene glycol) monomethyl ether methacrylate]-modified hyaluronic acid hydrogel and studied for ocular chemical alkali burn. In vivo, exosomes were used as a carrier for miRNA 24-3p and applied topically to rabbit corneal epithelial wounds. The formulation promoted corneal epithelial wound-healing, as well as the inhibition of corneal fibrosis and keratitis. The ocu-miRNA 24-3p (miRNA 24-3p)-rich exosome-loaded hydrogel showed novel effects in promoting corneal wound closure in vivo in rabbit corneal epithelial cells [46].

### 6.2. Exosomes with Gene Therapy

Gene delivery has been viewed as an emerging therapy to treat corneal opacity by delivering genes of interest via low immunogenic EVs. Zhao et al. observed that exosomes loaded with c-Rel-specific siRNA, a siRNA targeting the c-Rel member of the NF-KB family involved in the regulation of inflammatory responses, can effectively accelerate the healing of both regular and diabetic corneal wounds in vivo [47].

A study conducted by Shojaati et al. examined the effects of MSCs from corneal stromal stem cells (CSSCs) and their secreted EVs/exosomes on corneal fibrosis and inflammation. CSSCs produced EVs 130–150 nm in diameter, which expressed surface proteins used to classify EVs (CD63, CD81, and CD9). However, the EVs themselves produced similar effects to the CSSCs, in regard to reducing visual scarring in murine models. This effect was achieved by decreasing the expression of the fibrotic genes Col3al and Acta2, reduced neutrophil infiltration, and restoration of the corneal tissue morphology. This study also examined the importance of the PDCD6IP (Alix) gene in upregulating regenerative function and reducing corneal scarring, which was discerned by utilizing siRNA to knock down the mRNA encoding for Alix. The group’s in vivo studies determined that CSSC-derived EVs reduced corneal scarring, preserved the cornea’s morphology, and reduced early neutrophil infiltration [48].

### 6.3. Stem Cell-Derived Exosomes for Corneal-Related Investigations

Currently, the majority of exosomes incorporated into ophthalmic drug delivery systems are produced from mesenchymal stem cells (MSCs). These hematological cells, often sourced from bone marrow and birth-associated tissue types, differentiate into many types of connective tissues in the body, including cartilage, bone, skeletal muscle, and adipose tissue [21]_._ MSCs are a widely studied source of exosomes due to their simple isolation, abundance, and ability to be easily biochemically modified, as well as their ability to produce enormous amounts of exosomes [21]. In vitro and in vivo studies on the eye have shown that MSCs are able to differentiate into keratocytes and other corneal stromal cells. Keratocytes, mesenchymal-derived cells of the corneal stroma, can revert to their repair phenotype and respond to ocular injury. Unlike stem cells, using only the exosomes from stem cells for regenerative medicine eliminates some of the biosafety risk that is associated with stem cell therapy [49]. Carter et al. examined the effects of the bone marrow-derived MSC paracrine factors on corneal fibroblast cells in vitro and ex vivo. One of the experimental groups included MSCs co-cultured with electrospun fibers, which displayed a faster wound closure rate than other experimental groups in vitro. In an ex vivo study, MSCs alone and the MSC-electrospun fiber co-culture exhibited similar rates of wound closure and reduced corneal opacity but still surpassed that of the control group. However, the MSC-electrospun fiber co-culture did exhibit higher cell viability [50].

Exosomes are also recognized as a vital player within every cell’s secretome, which is a term used to describe the whole of a cell’s secreted messenger substances. Secretomes include a wide array of EVs, such as microvesicles, membrane particles, peptides, and small proteins, like cytokines [51]. The secretomes of MSCs have been widely studied, with many promising results regarding their anti-inflammatory and anti-angiogenic properties. A study conducted by Kyung-Sun et al. [41] examined the effects of the MSC secretome by utilizing topically applied MSC-loaded PEG–collagen hydrogels on alkali-burned rabbit corneas in an ex vivo model. The groups containing MSCs alone and MSCs encapsulated in hydrogel both accelerated wound closure, reduced stromal haze formation, reduced corneal opacity, and facilitated epithelial healing. However, the group containing the MSCs encapsulated in hydrogel produced slightly more remarkable results regarding reductions in corneal haze. The delivery of MSCs and their secreted paracrine factors alongside novel biomaterials describes the effects of a lyophilized MSC secretome delivered via a viscoelastic gel carrier on both mechanical injury and alkali burn injury to the cornea both in vitro and in vivo. The in vitro study determined that the formulation increased HCEC proliferation, which was exemplified by increases in cell metabolic activity and DNA concentration. For an in vivo study utilizing a mechanical wound model in rat corneas, the MSC secretome viscoelastic gel increased the rate of re-epithelialization. However, in the in vivo studies utilizing a corneal alkali burn model, the gel enhanced re-epithelialization while also reducing scar formation and hemorrhage. For both the in vitro and in vivo studies, upregulation of the CD44 receptor was observed, suggesting that the effects of the formulation may be dependent upon interactions with the receptor [52]. Another study examining the effects of secretome derived from TNF-a stimulated MSCs in an in vivo mouse model designed to replicate corneal limbal stem cell deficiency (LSCD) induced by an alkali burn injury. LSCD is a condition characterized by corneal conjunctivalization, vascularization, recurrent corneal erosions, and corneal opacity and/or blindness. By pre-treating the MSCs with TNF-a prior to administration, the group stimulated the MSC’s immunosuppressive function involving a variety of soluble factors. A concentrated, conditioned medium containing the MSC secretome was applied topically to the eye for 4 weeks and suppressed alkali-induced epithelial cell damage, as well as corneal neovascularization and inflammation [53]. An et al. examined the effects of the MSC secretome and the specific role of exosomes in induced wound-healing effects. This study emphasized the importance of a MSC’s secreted cellular messengers in their overall wound-healing effect. When two experimental groups were compared, one including MSC EVs and one considered to be EV-depleted, the group still possessing the ability to secrete paracrine factors had an increased rate of cell proliferation and a dose-dependent promotion of wound-healing in vitro. Upon conducting the in vivo studies, this same dose-dependent promotion of wound-healing was observed in the EV-proficient group, which was determined to be dependent upon the EV fraction utilized [54].

**Table 1:** Exosome-based cornea-related investigations.

Exosome Source	Type of Cornea Wound	Method of Research	Results	Ref
Human corneal epithelial cells (hCECs), corneal fibroblasts (hCFs), corneal endothelial cells (hCEnCs)	Corneal injury	In vitro—Exosomes derived from the three different corneal cell types were characterized using TEM, DLS, and Western blot analyses. Uptake of the three different exosome types into hCECs was examined using immunofluorescence staining; effects on hCEC proliferation was examined using immunofluorescence staining in combination with Ki-67 assay. To determine how the exosomes affected signal transduction pathways, phosphorylation levels of protein mediators were examined using the human phosphor-kinase proteome profiler array. Gene expression of hCECs, hCFs, and hCEnCs post-treatment were analyzed using robust multi-array analysis (RMA). Gene Interaction Network Analysis was used to predict the biological functions affected by exosomes in HCECs and hCFs.	Exosomes from all three cell types increased the rate of wound closure of scratch-wounded HCECs. Profiling of activated kinases show that corneal cell-derived exosomes activate signal transduction mediators for the HSP27, STAT, β-catenin, GSK-3β and p38 pathways. The mean gene expression profile of hCECs cultured with exosomes would likely promote cell proliferation and migration while also reducing differentiation.	[27]
Induced pluripotent stem cell-derived mesenchymal stem cells (iPSC-MSCs) Modification: exosomes derived from iPSC-MSCs transfected with miR-432-5p were combined with a thermosensitive, chitosan-based hydrogel (CHI hydrogel)	Unspecified corneal disease	In vitro—iPSC-MSC-Exos were isolated via centrifugation and characterized via TEM, DLS, and Western blot assay. CHI hydrogel and exosome-loaded CHI hydrogel were subjected to rheological studies and release properties were analyzed using a BCA assay. Biocompatibility studies were conducted via co-culture with HCECs and MSCs to examine effects on proliferation and mRNA contents via CCK-8 assay and RT-qPCR-based miRNA microarray analysis, respectively. In vivo—An anterior lamellar damage model was established in 42 rats using an operating knife. Ofloxacin eye drops were used for three days post-op. Corneal epithelial defect was monitored using fluorescent staining paper, and clarity was monitored via a slit-lamp microscope. The rat model was used for histological analysis via H&E staining, protein expression via immunofluorescence staining, and detection of miRNA expression of related genes via microarray analysis.	iPSC-MSC-Exos were found to encapsulate miR-432-5p, which modulates collagen biosynthesis in corneal stromal stem cells. miR-432-5p prevented ECM deposition via repression of TRAM-2. The formulated exosome-loaded CHI hydrogel allowed for sustained release of exosomes and encouraged regeneration of the corneal epithelium and stromal layer. Stroma regeneration was achieved via downregulation of mRNA expression encoding the three most enriched collagens. Additionally, in vivo experiments confirmed the ability of iPSC-MSC-Exo-loaded CHI hydrogel reduced corneal scarring.	[45]
Human mesenchymal stromal cells (hMSCs) Cargo: c-Rel-specific siRNA	Regular and diabetic corneal injury	In vitro—Effects of loaded-exosomes were examined via immunofluorescence staining and flow cytometry. In vivo/ex vivo—c-Rel-deficient and wild-type mice were used; scraping was used to cause a 2.5-mm diameter injury; wounds were staining with fluorescent dye and examined under a slit lamp microscope at specific time points and production of inflammatory cytokines was examined using ELISA. Both regular and diabetic corneal injury mouse models were used to examine the effects of topical treatment with c-Rel-specific siRNA, either encapsulated with nano-polymers or exosomes, effects of treatment were examined using rates of wound closure.	Expression of c-Rel and its inflammatory targets are increased with corneal injury in mice; c-Rel-deficient mice exhibit accelerated corneal wound-healing. Topical treatment of the corneal surface with exosomes or nano-polymers loaded with c-Rel-specific siRNA can accelerate corneal wound-healing. However, this study shows that siRel-loaded exosomes show better efficacy than nano-polymers.	[47]
Mesenchymal stem cells from corneal stromal stem cells (CSSC) Cargo: nonmammalian miR159a	Corneal inflammation and fibrosis after corneal wound	In vitro—EVs from CSSCs were characterized via estimating protein concentration via UV absorbance, TRPS, immunoblotting, TEM, and flow cytometry. EVs were labeled via fluorescent tag and loaded with miR159a via incubation with PBS. To determine if EVs transfected miRNA to HCECs, qPCR and RNA sequencing were used. In vivo/Ex vivo—A corneal wound model was established in mice using an Algerbrush. Two weeks after corneal debridement, eyes were collected and subjected to imaging, determination of gene expression via RT-PCR, immunoblotting, and neutrophil myeloperoxidase assay.	CSSC EVs labeled with fluorescent successfully fused with HCECs and CSSCs in vitro to transfer miRNA causing Alix knockdown. EVs with Alix knockdown exhibited lower amounts of miRNA, were ineffective at reducing fibrotic scarring, and lost regenerative function. In vivo, EVs from CSSCs reduced fibrotic scarring, and stimulated regeneration of stromal tissue; it is thought these effects result from CSSC EVs ability to reduce expression of fibrotic genes, block neutrophil infiltration, and restore normal corneal tissue morphology.	[48]
Mesenchymal stromal/stem cells	Corneal epithelial wound	In vitro—Effects of the MSC-conditioned media (MSC-CM; containing the secretome and exosomes of MSCs without the MSCs themselves) and EV-depleted MSC-CM on HCECs and human corneal limbal epithelial cells was examined; HCEC viability was examined via trypan blue incubation with microscopic examination; Cell proliferation and cytotoxicity were determined by measuring cellular DNA content via fluorescence and LDH assay, respectively; HCLE cells underwent a cell scratch assay using a spinning disc confocal microscope; In vivo—Mouse model of corneal epithelial wound-healing was conducted using an Algerbrush; images illustrating effects on wound-healing on corneas were obtained after staining with fluorescent dye; intensity fold change of corneal fluorescein staining was examined.	In vitro studies using HCECs showed that MSC-CM increased cell proliferation in HCEC and HCLE cells, while EV-depleted MSC-CM showed lower cell proliferation in both cell lines. In vitro and in vivo experiments revealed MSC-CM promoted wound-healing in a dose-dependent manner, while exosome deprivation delayed wound-healing. Incubation period of MSC-CM also influences its therapeutic effects, showing 72H incubation is more effective than 48H. Stability evaluation showed that after one cycle of free-thawing, MSC-S is stable at 4 °C for up to 4 weeks. This study elucidated that MSC-CM is the active ingredient in the MSC secretome and improved corneal barrier and decreased corneal haze/edema.	[54]
Human MSCs derived from cadaver corneas	Non-specific corneal epithelial wound	In vitro—Human corneal epithelial cells (HCECs) were scratch-wounded and then treated with 1.0 × 10^5^, 1.0 × 10^6^, or 1.0 × 10^7^ exosome or phosphate buffered saline, (PBS) which served as a vehicle control In vivo—Exosomes were applied topically to mice corneas with 2-mm epithelial debridement wounds	Corneal MSCs (cMSCs) exosomes were successfully taken up by human cMSCs in vivo and in vitro cMSC exosome treated HCECs had improved corneal wound-healing compared to the control group	[55]
Saliva from healthy human subjects—commercially purchased salivary exosomes	Diabetic cornea and keratoconus	Ex vivo—Scratch and cell migration assays were performed over 48 h on primary human corneal fibroblast cells which were isolated from 4 donor human corneas	Higher concentrations of salivary exosomes with high anti-inflammatory biomarkers (CD-9, CD-63, CD-81) promoted wound-healing through late stages of disease when fibronectin was downregulated	[56]
Bone marrow mesenchymal stem cells (BM-MSCs)	Diabetic corneal epithelium wound/diabetic keratopathy	In vivo—Diabetic mouse models were established using streptozocin (STZ) and their central cornea was scratched. Exosomes were traced with PKH-26	Corneal epithelium healing rates in the experimental groups (subconjunctival injection of exosomes derived from BM-MSCs or subconjunctival injection of BM-MSCs) were significantly higher at 24, 48 and 72 h. There was no significant difference in healing rates between the two experimental groups	[57]
Human limbal mesenchymal stem cells (hLMSCs) Modification: hLMSCs were treated with various concentrations of melatonin (Mel) to induce exosome production	Corneal scarring	In vitro—The effects of exosomes procured from melatonin-treated hLMSCs (Mel-prExos) on cell viability was evaluated using the MTT assay; expression of miR-155 (associated with α-SMA expression), miR-29 (associated with TGFβ1/β3 expression), and PPARγ miRNAs and genes were compared between melatonin-treated hLMSCs and Mel-prExo-treated hLMSCs.	At 1 μM of melatonin and in the presence of Mel-prExos, TGFβ1 was expressed 0.001-fold, while TGFβ3 was expressed 0.6-fold. miR-29 expression was increased 38-fold in the control-Exo group compared to that of the control group. Melatonin and mel-prExos increased the expression of anti-fibrotic genes and miRNAs and promoted the pro-regenerative effects of naïve hLMSCs, as evidenced in increased TGFβ3 and PPARγ expression and the inhibition of TGFβ1.	[58]
Wharton’s jelly mesenchymal stem cells (WJ-MSC) Modification: WJ-MSC-Exos are combined in a solution with amniotic membrane extract (AME)	Corneal wounds	In vitro—Characterization of AME and WJ-MSC-Exos was conducted using DLS and SEM; Cytocompatibility was determined using MTT assay and alamarBlue cell proliferation assay; Actin cytoskeleton and nuclei staining was used to determine cellular morphology of adherent corneal keratocytes following AME/WJ-MSC-Exo treatment; wound-healing potential of the formulation was investigated with scratch and cell migration assays.	The presence of both WJ-MSC-Exos and AME synergistically enhance the proliferation of corneal keratocytes increased wound closure rates.	[59]
M1 macrophages Modification: M1 macrophages were conditioned with epidermal growth factor (EGF)	Ocular surface inflammation	In vitro—Proteome analysis, pathway analysis, and QT-PCR was conducted on M1 macrophages to determine if the derived exosomes carried inflammation-relieving signals. Ex vivo—Effects of the M1 and EGF-M1 exosome eye drops on inflammatory signals and vasculogenic factors were examined using immunofluorescence staining of pan-macrophage markers. In vivo—Absorption of exosome eye drops was investigated using immunofluorescence assays in a mouse model.	Exosomes derived from EGF-treated M1 macrophages had enriched proteomic profiles contributing to their ability to regulate the immune system and inflammation. When applied as eye drops in mouse corneas, the exosomes reduced inflammation and increased M2-related ARG1 expression. The effects of EGF-M1-Exo eye drops differed from that of M1-Exo eye drops in their ability to suppress IL-1B, IL-6, VCAM1, ICAM1, VEGFA, PDGFA, MMP2 and MMP9 gene expression, while causing activation of ARG1 gene expression.	[60]
Bone Marrow Mesenchymal stem cells (BMSCs) Cargo: miRNA-29b-3p agomir/antagomir	Inflammation secondary to corneal injury	In vitro—Exosome tracking on immortal HCECs (iHCECs) was performed via fluorescence assay; effects on cell migration, proliferation, and viability were examined using fluorescence assays, flow cytometry and TEM, and CCK-8 assay, respectively. In vivo—Exosome tracking on injured murine corneas was performed via fluorescence assay; the effects of Exos on corneal inflammation, haze production, gene expression, and autophagy were determined using fluorescein staining and bright field photographs, H&E staining, corneal haze grading, TEM, Western blot analysis, and RT-qPCR; effects of the exosomes on cytokine expression were examined using immunohistochemical analysis.	Compared to PBS, Exos-29b-agomir, Exos-29b-antagomir, and Exos-control all could produce therapeutic effects on corneal inflammation and fibrosis. Exos-29b-agomir, however, encapsulated large amounts of miR-29b-3p and had profound therapeutic effects on autophagy activation and corneal inflammation via inhibition of PI3k/AKT/mTOR pathways and inhibition of mTOR/NF-κB/IL-1β pathways, respectively. Exos-29b-agomir decreased expression of collagen type III, α-smooth muscle actin, fibronectin, and vimentin. Additionally, the overexpression of miR-29b-3p in Exos-29b-agomir prevented autophagy impairment and inflammatory injury in iHCECs.	[61]
Myeloid-derived suppressor cells (MDSCs) Modification: MDSCs were conditioned with rapamycin	Corneal allograft rejection	In vitro and Ex vivo—Determination of the active component contributing to Rapa-Exos anti-rejection effects, as well as the mechanism of action, were determined using RT-qPCR, slit-lamp images of corneal allografts, survival curves via the Kaplan-Meier approach, H&E staining, and ELISA. In vivo—The effects of Rapa-Exos on corneal allograft rejection were examined in a well-characterized murine corneal transplantation model using slit-lamp images of corneal grafts, histopathological examination via H&E staining, RT-qPCR, and immunofluorescence staining.	The Rapa-Exos derived from rapamycin-conditioned MDSCs exerted superior effects when compared to Exos derived from untreated MDSCs. In vitro assays revealed that the anti-rejection effects are due to functions of miR-181d-5p, which causes KLF6 knockdown. KLF6 knockdown resolves inflammation and prolongs survival of corneal allografts through immunosuppressive effects.	[62]
Adipose-derived mesenchymal stem cells (ASCs)	Corneal endothelial injury	In vitro—The effects of ASC-derived exosomes (ASC-Exos) on the regenerative capacity of human corneal endothelial cells was examined using cell viability and cell-cycle analyses; to examine the difference in expression of miRNAs between ASC-derived and human corneal endothelial cell-derived exosomes, RT-qPCR was performed; uptake, cell viability, cytotoxicity, and cell cycles of corneal endothelial cells treated with ASC-Exos was determined using fluorescence microscopy, CCK-8 assay, LDH assay, and propidium iodide staining with flow cytometry, respectively; To examine the effects of ASC-Exos on induced endothelial-to-mesenchymal transition (EMT) and mitophagy, Western blot analyses, immunofluorescence staining, and determination of mitochondrial membrane potential were performed, In vivo—ASC-Exos were introduced into rat corneal endothelial cells using electroporation after corneal injury was induced via cryoinjury. To determine the effects of ASC-Exos on rat corneal endothelium, fluorescence microscopy, TUNEL staining, and Alizarin Red S staining, Ki67 staining, immunofluorescence staining, was performed. Differential miRNA expression was determined via RT-qPCR.	ASC-Exos induced corneal endothelial cell proliferation and suppressed or protected against TGFβ- or H_2_O_2_-induced oxidative stress/senescence and induced EMT and mitophagy. In vivo studies demonstrated the wound-healing effects of ASC-Exos in rat corneal endothelial cells and protected those cells from cryoinjury and related damage. RNA sequencing analysis showed that the miRNAs expressed by ASC-Exos and human corneal endothelial cell-derived Exos influence lysine degradation, adherens junction, TGFβ, p53, Hippo, FoxO signaling, actin cytoskeleton regulation, and RNA degradation.	[63]
Human mesenchymal stem cells (MSCs)	Corneal scarring	In Vitro—Cellular uptake of MSC-Exos, as well as their immunomodulatory effects, were examined in both corneal stromal fibroblasts and myofibroblasts; cellular uptake was examined using fluorescence intensity and immunoassays were used to determine immunomodulatory effects; wound-healing effects were examined via a scratch wound assay on HCET cell culture, determined via rate of wound closure and fluorescein staining. Ex vivo—Doses of MSC-Exo were applied to epithelium-on and -off corneas to determine if a relationship exists between dosing and retention after topical administration, this was accomplished via use of a confocal laser scanning ophthalmoscope and fluorescent microscopy; immunohistochemistry, immunoassay and RT-qPCR were used to assess relative molecular changes and immunomodulatory effects in rat corneas after 5 days of topical administration, respectively. In vivo—Rat corneas underwent irregular phototherapeutic keratectomy to examine MSC-Exos effects on corneal stromal haze, which was determined via slit-lamp imaging, confocal microscopy, anterior segment-optical coherence tomography, and neovascularization score.	When compared to the PBS control group, the MSC-Exos treatment group had faster epithelial wound closure, lower corneal haze score, and reduced haze intensity during the follow-up period. Attenuation of corneal vascularization, based on CD31 and LYVE-1 staining, as well as reduced fibrosis as measured by fibronectin and collagen 3A1 staining was also observed in the MSC-Exo group. MSC-Exo treated corneas exhibited a regenerative immune phenotype characterized by higher infiltration of non-inflammatory immune cells (vs non-inflammatory immune cells), reduced levels of pro-inflammatory cytokines, and increased levels of anti-inflammatory cytokines. This formulation may alleviate corneal injury.	[64]
Bone marrow-derived mesenchymal stem cells (BMSCs)	Corneal alkali burn	In vitro—Isolated BMSC-Exos were identified via TEM, Western blot analysis, and NTA; exosome uptake was examined via dye staining and use of laser confocal microscope. HCEC proliferation and migration was examined after scratching wound assay and coculture with BMSC-Exos. The protein expression of p-MEK/MEK and p44/42 MAPK in HCECs was detected via Western blot analysis. In vivo/Ex vivo—An alkali burn model in mice was used via sodium hydroxide exposure. After treatment with BMSC-Exos via injection, pathological changes and protein expression in excised corneas were examined via H&E staining, immunofluorescence, and immunohistochemistry.	In vitro, BMSC-Exos caused dose-dependent enhancement of HCEC proliferation and migration; p44/42 MAPK pathway was activated by BMSC-Exos treatment, and its blocking of U0126 was partially responsible for their ability to enhance HCEC proliferation and migration. In vivo, BMSC-Exos injection reduced pathological changes inducing inflammation, and decreased upregulation of α-SMA and CD31, the proteins responsible for fibrosis and vascularization in corneal tissues, respectively.	[65]
Umbilical cord mesenchymal stem cells (HUMSCs) Cargo: miR-21	Corneal epithelial wound	In vitro—HUMSC small extracellular vesicles (HUMSC-sEVs) were identified via TEM, NTA, and Western blot analyses. HCEC proliferation post-treatment with HUMSC-sEVs were examined using CCK-8 and EdU assays. HCEC migration was evaluated using a scratch wound assay. The genes expressed by HCECs post-treatment with HUMSC-sEVs was examined via full-length transcriptome sequencing, RT-PCR, and Western blot analyses. In vivo/Ex vivo—A corneal mechanical wound model was established in rats via a unilateral corneal injury, rats received a subconjunctival injection of HUMSCs or HUMSC-EVs. Wound residual area was monitored using fluorescein staining and imaging via a slit lamp microscope. H&E staining alongside light microscopy was used to examine corneal structure and degree of re-epithelialization.	In vitro, HUMSCs were less effective when the release of exosomes was blocked, suggesting sEVs/exosomes play a vital role in wound-healing and HUMSC function. HUMSC-sEVs promote HCEC proliferation and migration. In vivo, HUMSC-sEVs promoted corneal epithelial wound-healing, as well as HCEC proliferation and migration. H&E staining revealed that corneas treated with HUMSC/HUMSC-sEVs regained a more regular arrangement and compact structure than those treated with PBS. After miR-21 transfection, beneficial effects of treatment were partially negated. HUMSC-sEVs may potentially enhance the recovery of corneal epithelial wounds via miR-21 transference, causing repression of PTEN expression, which in turn activate the PI3K/Akt signaling pathway in HCECs.	[66]
Human umbilical cord mesenchymal stem cells (hucMSC) Modification: used in combination with autophagy activators	Corneal injury	In vitro—hucMSC-Exos were isolated and identified via NTA, TEM, and Western blot analyses. The effects of treatment with hucMSC-Exos combined with autophagy regulators were examined in HCECs via cell viability assays, scratch assays, cell cycle assays and apoptosis assays. In vivo—The corneal injury mouse model was conducted using an AlgerBrush II corneal remover. The effects of hucMSC-Exos combined with autophagy regulators on mice were examined using corneal fluorescein staining, haze grades, H&E staining, immunohistochemical staining, TUNEL assay, TEM, Western blotting, and qPCR.	In vitro results indicate the hucMSC-Exos combined with the autophagy activator increased cell proliferation and migration capacity, in addition to positive effects on the cell cycle via upregulating the proportion of cells in the S phase and increased expression of proteins involved in proliferation. Combination treatment with autophagy activator reduced apoptosis in HCECs. In vivo, hucMSC-Exos with autophagy activator mitigated epithelial wounds and opacity in the stroma. Additionally, the combination treatment reduced levels of apoptotic and inflammatory markers via activation of the AMPK-mTOR-ULK1 signaling pathway.	[67]
Mesenchymal stem cells (MSCs)	Corneal allograft rejection	In vitro—MSC-derived exosomes were collected, and then characterized using electron microscopy and Western blot analysis. In vivo—Using the Wistar-Lewis rat corneal allograft rejection model, the rats were divided into four groups with six rats in each group; each group received a different concentration of MSC-exo via two subconjunctival injections: one was given postoperatively, and the other was given two days post-op. All exosomes administered via injection were labeled via PKH26 for tracking. Rats were observed post-op day three via a slit-lap microscope and given an opacity score. Ex vivo—On day 10, rat eyes were excised and subjected to gene expression array, histopathological examination, immunohistochemistry. staining and photography for exosome tracking. Grafts taken from subjects were examined via qPCR. Spleens and lymph nodes were also collected, and monocytes were examined via flow cytometry.	The isolated exosomes expressed the desired exosome biomarkers and were detected in both the cornea and anterior chamber two hours post-injection. The rats that received the 10 μg injection exhibited increased graft survival time, and ex vivo analyses showed inhibition of CD4+ and CD25+ T cell infiltration. In all groups receiving MSC-Exo injection, ex vivo analysis of excised grafts showed reduced levels of the pro-inflammatory cytokines IFN-γ and CXCL11.	[68]
Mesenchymal stem cells (MSCs) and blood serum (SER)	Corneal endothelial dystrophy	In vitro—MSC-EVs and SER-EVs (used as a control; thought to possess pro-angiogenic activity) were isolated and characterized using NTA, super-resolution microscopy, MACSPlex flow cytometry, and fluorescent staining. Endoplasmic stress was induced in HCECs via serum deprivation and tunicamycin application; the presence of ER stress biomarkers was determined using RT-PCR, and ER stress-related protein phosphorylation was examined via Western blot analysis. To determine if HCECs expressed apoptotic markers post-treatment, a phosphatidylserine assay was conducted using Annexin V and 7-AAD. To determine if MSC-EV’s miRNA were successfully transferred to HCECs, a Funrich analysis and RT-PCR were performed.	In the in vitro model of corneal dystrophy, the effects MSC-EVs were compared with those of SER-EVs. SER-EVs exhibited minimal or absent effect on modulating endoplasmic reticulum stress and/or apoptosis. MSC-EVs caused downregulation of genes causing endoplasmic reticulum stress in an endoplasmic reticulum stress model in HCECs. MSC-EVs upregulated the Akt pathway, while also decreasing activation of apoptotic markers. The therapeutic effects of MSC-EVs is likely linked to the transfer of endoplasmic reticulum-stress targeting miRNAs to corneal endothelial cells.	[69]
Induced pluripotent stem cells (iPSCs) and mesenchymal stem cells (MSCs)	Corneal epithelial defect	In vitro—Characterization of exosomes derived from IPSCs and MSCs was conducted using NTA, TEM, and Western blot analyses. To assess cellular uptake of IPSC-/MSC-Exos by HCECs, immunofluorescence staining was employed. The effects of IPSC-/MSC-Exos on HCEC was evaluated using apoptotic assays, live-dead cell determination, scratch wound assay, and CCK-8 cell viability assay. The effects of the IPSC-/MSC-Exos on the HCEC cell cycle were examined using flow cytometry, immunofluorescent staining, RT-qPCR, and Western blot analyses. Ex vivo—Rat eyeballs were harvested at 48H and underwent histological analysis with H&E staining. In vivo—AlgerBrush was used to establish a corneal epithelial defect model in rats. To assess cellular uptake of IPSC-/MSC-Exos after topical administration, immunofluorescent staining and TEM were conducted. The effect of IPSC-/MSC-Exos on corneal epithelial defect healing was monitored every six hours over a 48H time period using fluorescein staining with slit lamp, observing healing rates, and monitoring of central corneal thickness as measured by AS-OCT at 24 and 48H.	In vitro, IPSC-Exos had a more beneficial effect on HCEC proliferation, migration, cell cycle promotion, and inhibition of apoptosis. Both IPSC- and MSC-Exos promoted cell regeneration by increasing upregulation of cyclin A and CDK2, which caused HCECs to enter S phase from G0/G1 phase. In vivo, both exosome types accelerated corneal wound-healing through modulation of cellular metabolism; when compared to a control group, IPSC-/MSC-Exos nearly completely restored corneal integrity. However, iPSC-Exos exhibited a much stronger therapeutic effect on accelerating wound-healing and re-epithelialization of corneal injury. This study suggests that increased therapeutic efficacy of IPSC-Exos may be attributed to their increased expression of proteins shared with their source cell line.	[70]
Adipose-derived stem cells (ADSCs)	Corneal fibrosis	Ex vivo—ADSCs and ADSC-Exos were isolated from rabbit adipose tissues and characterized using flow cytometry, NTA, and Western blot analyses. Rabbit corneal keratocytes were isolated and grown for 7 days in an FBS-containing medium (FBS induces differentiation of keratocytes into myofibroblasts). Rabbit corneal keratocytes were incubated with both ADSCs and ADSC-Exos, and then the effects of ADSC and ADSC-Exos were examined using CCK-8 cell viability assay and Western blot analysis. The effects of ADSC-Exos on the differentiation of keratocytes into myofibroblast investigated using QRT-PCR, Targetscan analysis, dual luciferase reporter assay, and Western blot analysis.	Keratocytes grown in FBS-containing medium differentiated into myofibroblasts by increasing HIPK2 kinase expression and activity. ADSCs and ADSC-Exos inhibited FBS-induced differentiation of keratocytes into myofibroblasts causing HIPK2 knockdown due to their miR-19a content. Target scan analysis confirmed that the HIPK2 3′UTR is the direct binding target of miR-19a.	[71]
Aqueous humor (AH)	Unspecified corneal wound	In vitro—Human immortalized keratinocyte cells (HaCaT) in a scratch wound assay model were used to investigate the effects of AH-EVs versus the effects of EVs derived from mesenchymal stromal stem cells (MSC-EVs) on viability, proliferation, and wound-healing. Ex vivo—AH was obtained from 10 patients with cataract who were undergoing surgical pharmacoemulsification and insertion of intraocular lenses. The EVs obtained from AH were characterized using NTA, electron microscopy, super resolution microscopy, and bead-based cytofluorimetry.	AH-EVs had a mean size of ~100 nm and expressed tetraspanins, such as CD9, CD63, and CD81, that are used to classify EVs. Co-expression of these tetraspanins was confirmed by super resolution microscopy. Confirmation of AH-EV expression of mesenchymal, stem, epithelial, and endothelial cell markers was determined via cytofluorimetric analysis. In the scratch wound model using HaCaT cells, the effects of AH-EVs were associated with faster wound-healing, as well as increased cell viability and proliferation.	[72]
Immortalized Human Corneal stromal stem cells (imCSSCs)	Corneal inflammation and fibrosis	In vitro—imCSSCs were grown in a conditioned medium, and EVs were isolated from the imCSSC line via a Total Exosome isolation kit and ultracentrifugation. EVs were cultured with trehalose dissolved in PBS prior to being lyophilized using liquid nitrogen. After snap freezing, EVs were stored for four weeks then evaluated using DLS and TEM. After storage, retained EV markers were determined using Western blot, fluorescent staining, flow cytometry, RT-PCR, and RAW cell differentiation/anti-inflammatory assay. Anti-fibrotic properties of EVs post-storage were examined using immunofluorescence staining and RT-PCR.	EVs stored at −80 °C were also lyophilized with trehalose most closely mimicked their pre-storage morphology; EVs maintained normal protein expression. EV samples that were lyophilized without trehalose exhibited lower particle concentrations, slower recovery rate, and decreased protein concentrations; although, this was remediated via addition of trehalose. Regardless of storage condition, all EV samples reduced inflammation and inhibited expression of fibrotic markers.	[73]

## 7. Clinical Trials

Human-derived exosomes have more detailed findings in clinical trials compared to plant-based exosomes, though both types of investigation have been documented in clinical studies. A prospective clinical trial on dry eye was conducted to determine if miR-204-loaded exosomes may relieve graft-versus-host disease-associated (GVHD) dry eye disease, utilizing exosomes derived from MSCs. The results showed 28 eyes with refractory GVHD dry eye disease exhibited substantial relief after MSC-exo treatment, showing reduced fluorescein scores, longer tear film breakup time, increased tear secretion, and lower OSDI scores. These exosomes were administered as eye drops and suppressed inflammation while also improving epithelial recovery in both mice and humans. A miRNA called miR-204 is the main immunomodulatory cargo in MSC-exo. MiR-204 specifically targeted the interleukin-6 receptor (IL-6R) to inhibit the expression of the IL-6/IL-6R/Stat3 process, causing inflammatory M1 macrophages to change to the immunosuppressive M2 phenotype on the corneal surface [74].

NCT04213248 aims to determine whether umbilical mesenchymal stem cell (UMSC)derived exosomes could alleviate dry eye symptoms in patients with chronic graft-versus-host disease. In this study, the effect of UC-MSC-Exo will be tested on 27 participants between the ages of 18 to 70 years. Participants will be administered artificial tears for 2 weeks, followed by UC-MSC-Exo at 10 μg/drop four times a day for 2 weeks. Participants will be followed up on for 12 weeks to measure the dry eye progression. The primary endpoint will be a change in Ocular Surface Disease Index (OSDI) score, and secondary endpoints include tear secretion, tear break time, areas stained by fluorescent, ocular redness, tear meniscus, and best-corrected visual acuity post-treatment [75].

In a phase II clinical trial (NCT03687632), the effects of a multi-cytokine-containing, novel secretome formulation were evaluated for the treatment of persistent corneal epithelial defects caused by a wide array of underlying diagnoses. The secretome itself was derived from amnion-derived multipotent progenitor (AMP) cells and was administered via an ophthalmic drop formulation. All 12 patients evaluated in the study showed re-epithelialization and, for 41.7% of the patient population, total wound closure by the end of a 28-day treatment. Preclinical in vitro and in vivo studies of the formulation, dubbed ST266, also examined the anti-inflammatory and neuroprotective properties of the formulation [76].

## 8. Future Perspective, Opportunities, and Challenges

With continued advancements in exosome investigations, it may assume a pivotal role in corneal disorders and injuries and in the future of regenerative ophthalmology. Here, we summarize the recent, ongoing, and continued perspectives on ocular-based exosomes drug delivery in multiple directions. Advanced exosome engineering and customization: Molecular engineering is expected to enable the exact tailoring of exosome cargo to target specific ocular tissues. Methods such as surface modification or functionalization may allow exosomes to transport elevated levels of anti-fibrotic drugs, growth factors, or particularly miRNAs, thus amplifying their healing effects [77,78,79]. Integration with stem cell and gene therapy: Exosome-based delivery systems offer a precise and efficient method for gene modulation, enhancing regenerative outcomes in corneal and other ocular tissues. These engineered exosomes encapsulate gene-editing tools, like CRISPR-Cas9 [80] and siRNAs, facilitating targeted delivery and overcoming traditional bioavailability issues [81]. Exosomes functionalized with RNA-binding proteins or hybridized with liposomes further improve the delivery of genetic material, enabling effective gene silencing and editing [82]. This approach highlights exosomes’ potential as powerful carriers for targeted gene therapy in regenerative medicine. Personalized exosome therapies: Exosome-based therapy holds promise for personalized medicine based on each patient’s distinct profile. Thus, analyze each corneal condition individually and tailoring exosome therapy for diverse patient needs to advance personalized medicine in ophthalmology [83,84]. Investigation of alternative exosome sources: Increasing studies on exosomes derived from different cell types, such as plant-based or non-human animal sources, may reveal novel characteristics or improve therapeutic outcomes. Accordingly, adequate investigations are required to ensure compatibility and safety, immune responses, and lowering inflammation in the eye from distinct sources [85]. Clinical translation and immunogenicity: In lieu of encouraging preclinical findings, exosomes’ immunogenic profiles for corneal applications are under investigation, and future studies ought to concentrate on the safety of exosomes in the immune-privileged ocular environment, specifically regarding the exosomes’ origin (e.g., human versus non-human sources) and possible long-term immunological reactions [83,86]. Regulatory challenges in exosome and EV therapy approval: The existing regulatory framework for exosomes and EV therapeutics encounters obstacles due to the absence of standardized rules, resulting in uncertainty in classification and assessment by organizations such as the FDA and EMA [87]. EVs fall between categories like biological products and cell therapies and can be mingled with drug delivery systems, delaying the regulatory guidelines [88]. Moreover, the intrinsic variability of EVs, arising from diverse cell origins, sizes, and cargo compositions presents further challenges in complying with regulatory requirements for product characterization and batch uniformity [89]. The variability in EV preparations can affect treatment efficacy and reproducibility, underscoring the requirement for generally agreed on protocols establishing practical standards for EV characterization, safety, and efficacy assessment [87].

## 9. Concluding Remarks

Exosomes are promising nanovesicles and can act as therapeutic active components alongside drugs. Their capacity to administer bioactive chemicals directly to specific corneal cells provides a non-invasive and biocompatible alternative to conventional treatments, particularly for diseases where the existing therapies are inadequate. Thus, they are recognized as promising next-generation therapeutics due to demonstrated safety and efficacy in preclinical models and early-stage clinical trials. Similarly, exosomes have the potential to serve as novel carriers for cornea-targeted drug delivery because of their low toxicity, ability to carry materials from one cell to another, adaptability with biological tissues, non-immunogenicity, and specific targeting of different cells. Here, we have highlighted some of the key advances of EVs in corneal treatment, repair, and regeneration, such as exosomes facilitate corneal healing, including controlling inflammation, encouraging cell proliferation, and inhibiting scarring. Given the complexity of the corneal healing cascade and immunogenicity of the eye, there will be many future EV-related discoveries together, unveiling the exciting benefits of exosomes-based therapeutic platforms, and further investigations are required for clinical translational challenges.

## Data Availability

Data sharing is not applicable.
